# Identification of a cancer stem cell-like side population in the HeLa human cervical carcinoma cell line

**DOI:** 10.3892/ol.2013.1607

**Published:** 2013-10-07

**Authors:** KEFANG WANG, JIANFANG ZENG, LIJING LUO, JIAXIN YANG, JIE CHEN, BIN LI, KENG SHEN

**Affiliations:** 1 Department of Obstetrics and Gynecology, Beijing Anzhen Hospital, Capital Medical University, Beijing 100029, P.R. China; 2 Department of Obstetrics and Gynecology, Peking Union Medical College Hospital, Chinese Academy of Medical Sciences, Beijing 100730, P.R. China; 3 Department of Pathology, Peking Union Medical College Hospital, Chinese Academy of Medical Sciences, Beijing 100730, P.R. China

**Keywords:** cervical carcinoma, HeLa cell line, side population cells, cancer stem cells, CD133

## Abstract

The present study aimed to identify the stem cell characteristics of side population (SP) cells sorted from the widely-used HeLa human cervical carcinoma cell line. The SP cells were sorted from the HeLa cell line using fluorescence-activating cell sorting (FACS). Stem cell characteristics of the SP cells, including proliferation, self-renewal, differentiation and the ability to form xenografts, were investigated *in vitro* and *in vivo*. The SP cells demonstrated strong tumorigenesis following *in vivo* transplantation into five to six-week-old female Balb/c mice. The SP cells were observed to be more resistant to chemotherapy and radiotherapy compared with non-side population (NSP) cells. A higher expression of CD133 was observed in the SP cells compared with the NSP cells following FACS. The results demonstrated that the SP cells from the HeLa human cervical carcinoma cell line exhibit stem cell characteristics *in vitro* and also have a strong ability to form tumors *in vivo*. The cell surface marker CD133 may serve as a potential molecular marker for the identification of cervical cancer stem cells (CSCs).

## Introduction

Cancer stem cells (CSCs) are important as they are capable of self-renewal, differentiation and the maintenance of tumor growth and heterogeneity. Studies suggest that CSCs are not only responsible for tumorigenesis, but that they also contribute to tumor recurrence and, in certain cases, resistance to cancer therapy (1). CSCs have been isolated from several human tumors that express markers for putative normal stem cells, including leukemia (2) and breast (3), brain (4), prostate (5) and ovarian (6,7) cancers. However, this research has been impeded by the lack of distinct molecular markers for CSCs.

The use of the Hoechst 33342 dye for identifying and isolating CSCs as a side population (SP) overcomes the phenotypical marker barrier and provides a functional marker (8). SP cells have been identified in various cell lines that have been generated from hepatocellular liver cancer (9), nasopharyngeal carcinoma (10) and ovarian cancer (11). These findings have demonstrated that SP cells exhibit stem cell characteristics and may be enriched as a stem cell population. Additionally, this method may aid in identifying more effective CSC markers by comparing the expression profiles of SP and non-side population (NSP) cells, and it may ultimately play a crucial role in the establishment of targeted cancer therapies.

Although this field is rapidly advancing, only a small number of published studies have examined the role of SP cells in human cervical cancer. Cervical cancer is the second most common cancer, after breast cancer, for females worldwide and it is also one of the most serious diseases that threaten female health. Although traditional surgery, radiotherapy and combined treatments have obtained good results for early-stage cervical squamous cell carcinoma, the results from patients who have been treated for cervical adenocarcinoma, which is an advanced and recurrent cervical cancer, are not favorable. The five-year survival rate is 30–50% for patients with stage III cervical carcinoma and only 5–15% for patients with stage IV disease. The five-year survival rate for patients with local recurrence and distant metastasis is ~10%. Thus, studying the CSCs in cervical cancers in order to understand their role in metastasis, recurrence and resistance to cancer therapy, is significant for identifying novel and more specific therapeutic approaches. The HeLa cell line is a commonly studied cervical adenocarcinoma cell line with a high capacity for malignancy (12). The present study aimed to identify the prevalence of SP cells in the HeLa cell line and to evaluate the stem cell-like subpopulation of the HeLa cancer cells. The subsequent results should aid in forming a foundation for the design of future therapeutic strategies for cancer patients.

## Materials and methods

### Cell culture

The HeLa human cervical adenocarcinoma cell line infected with HPV18 was obtained from Professor Chen (Department of Pathology, Peking Union Medical College Hospital, Chinese Academy of Medical Sciences, Beijing, China) and maintained in the Department of Gynecology and Obstetrics, Peking Union Medical College Hospital, Chinese Academy of Medical Sciences. The cells were cultured in DMEM media (Gibco, Carlsbad, CA, USA) supplemented with 10% FBS (Gibco), 100 U/ml penicillin and 100 μg/ml streptomycin (Gibco) at 37ºC in a 5% CO_2_ humidified incubator.

### Cell sorting

The cells that were in the logarithmic phase of growth were analyzed by fluorescence-activating cell sorting (FACS; FACS Diva Option; Becton Dickinson, Mountain View, CA, USA). The cells were harvested using 0.25% trypsin (Gibco), washed twice with PBS solution (Hyclone, Logan, UT, USA) and resuspended in DMEM at a concentration of 1×10^6^ cells/ml. The cells were then incubated with 5 μg/ml Hoechst 33342 dye (Sigma-Aldrich, St. Louis, MO, USA) for 120 min in the dark at 37ºC and mixed at 15 min intervals. The cells were washed twice with pre-cooled PBS and centrifuged at 1,000 × g at 4ºC. The cells were counterstained with 1 μg/ml propidium iodide (Sigma-Aldrich) in the dark and sorted by FACS using a dual-wavelength analysis. Hoechst 33342 is extruded from cells using a verapamil-sensitive ABC transporter, which is a calcium channel antagonist. Therefore, a subset of the cells were incubated with verapamil (50 μmol/l) for 30 min at 37ºC prior to adding Hoechst 33342, in order to determine whether the verapamil treatment was able to block the fluorescent efflux from the HeLa SP cells. The SP and NSP cells were collected for further experiments.

### Cell proliferation

Freshly sorted SP and NSP cells were cultured at a density of 400 cells per 35-mm culture dish in DMEM at 37ºC in a 5% CO_2_ incubator. The growth of the cells was monitored and images of the cells were captured at two, five, seven and eight days.

In parallel, the freshly sorted SP and NSP cells were seeded in 96-well plates at a density of 1,000 cells/well in 0.2 ml DMEM and cultured at 37ºC in a 5% CO_2_ incubator to observe the growth rate. The culture medium was removed following a 24-h incubation period and 0.2 ml 3-(4,5)-dimethylthiahiazo(-z-y1)-3,5-di-phenytetrazoliumromide (MTT) solution (final concentration, 0.5 mg/ml; Sigma-Aldrich) was added to four of the wells. The cells were incubated for 4 h and the medium was replaced with 0.15 ml DMSO. The plates were shaken for 10 min. A photometer (lQuant; Bio-TEK, Winooski, VT, USA) was then used to measure the absorbance of the cells at 490 nm every 24 h for eight days. The experiments were repeated three times and a cell growth curve was produced using the average values.

### Colony-forming cell assays

The freshly sorted SP and NSP cells were counted, plated in triplicate at a density of 250 cells per well in six-well plates and cultured with DMEM for ~10 days. Following the expansion of the majority of the cell clones to >50 cells, the cells were washed twice with PBS, fixed in formaldehyde for 15 min and stained with crystal violet for 20 min at room temperature. Subsequent to washing the stain out, the numbers of colonies that contained >50 cells were counted and the results were compared. The clone formation efficiency (CFE) was the ratio of the clonal cell number to the plated cell number. The results represent the average CFEs of three experiments.

### Long-term differentiation of SP and NSP cells

The freshly sorted SP and NSP cells were cultured in DMEM in a humidified 5% CO_2_ incubator at 37ºC. The differentiation assay was performed at three weeks post-incubation, when the cells reached the target cell number. The cultured SP and NSP cells were stained with Hoechst 33342 and analyzed using FACS to quantify the proportion of SP cells and to determine the differentiation ability of the two subpopulations. Following the second sorting, the SP cells were cultured in DMEM. At approximately two weeks post-culture, the SP and NSP cells were stained and analyzed using FACS for the third time to determine whether the SP cells were enriched through repeated cell sorting.

### Tumor formation assay

Female five to six-week-old Balb/c mice were purchased from the animal institute of the Chinese Academy of Medical Science (CAMS, Beijing, China) and Peking Union Medical College (PUMC, Beijing, China) and maintained in the barrier system of a specific pathogen-free (SPF) environment. Approval for the study was obtained from the animal care committee of CAMS and PUMC. A total of 24 Balb/c mice were randomly divided into eight groups containing three animals each. For the assay groups, 1×10^3^, 1×10^4^, 1×10^5^ and 1×10^6^ freshly sorted SP and NSP cells were suspended in 200 μl PBS and injected into the axillary fossa of the Balb/c mice. The three mice within each group were injected with a different cell type and number of cells. The mice were monitored twice weekly for the formation of palpable tumors and sacrificed at eight weeks post-transplantation in order to assess tumor formation.

### Radiation and drug sensitivity assays

The SP and NSP cells were exposed to X-rays in order to determine the differences in radiation sensitivity. A total of 100 freshly sorted SP and NSP cells were seeded per well in a 24-well plate with each cell type in six wells. Of the total wells, three of each cell type were irradiated with 8 Gy of X-ray (1,000 cGy/min using a 12×6-cm irradiation field) the day after seeding, and the cells were then cultured. The remaining three wells from each cell group that were not exposed to X-rays were cultured as matched controls under normal conditions. The cells were stained with crystal violet at three weeks post-irradiation and the clone number was determined, which reflected the ability of the cells to survive irradiation.

In parallel, freshly sorted SP and NSP cells were seeded in 96-well plates at a density of 1,000 cells/well in 0.2 ml DMEM and cultured at 37ºC in a 5% CO_2_ incubator. The culture medium was removed 24 h later and medium containing 12.5, 25, 50, 100 or 200 μg/ml cisplatin (8) was added. Each cisplatin concentration was used in quadruplicate. Additionally, one group was used with a concentration of 0 μg/ml as a blank control and three groups were incubated in DMEM only. All the cells were cultured in 0.2 ml DMEM at 37ºC in a 5% CO_2_ incubator for 24 h. The culture medium was removed and 0.2 ml MTT was added to each well. The cells were incubated for 4 h and the medium was replaced with 0.15 ml DMSO. The plates were shaken for 10 min. The absorbance was measured at 490 nm in a photometer within 30 min. The formula that was used for the determination of the cell inhibition ratio is as follows: Cell inhibition ratio = no-cisplatin control group A value − (experimental group A value/no-cisplatin control group A value).

### CD133 and CD44 expression in SP and NSP cells

HeLa cells and freshly sorted SP and NSP cells were cultured in DMEM in a humidified 5% CO_2_ incubator at 37ºC. At approximately one week post-incubation, the cells were treated with trypsin, washed and resuspended in 100 μl PBS solution. PE-labeled CD133 antibodies and FITC-labeled CD44 antibodies (Cell Signaling Technology, Inc., Boston, MA, USA) were added and the cells were incubated in the dark for 1 h. The cells were washed twice in PBS and analyzed using FACS.

### Statistical methods

Student’s t-test or a one-way ANOVA were used where appropriate. The analyses used the SPSS 10.0 statistical software (SPSS, Inc., Chicago, IL, USA). P<0.05 was considered to indicate a statistically significant difference. Data are expressed as the mean ± SD from at least three independent experiments.

## Results

### Cell sorting

**Figure 1:**
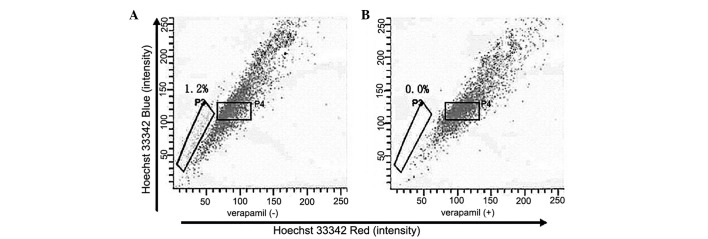
Hoechst 33342 staining of several human cervical cancer cell lines, analyzed using flow cytometry. (A) SP (P3 gate) and NSP (P4 gate) cells that were sorted from the HeLa cell line in the absence of verapamil. (B) The fraction of SP cells decreased to 0% in the presence of inhibition by verapamil. SP, side population; NSP, non-side population.

Subsequent to excluding the dead cells and cellular debris based on scatter signals and propidium iodide fluorescence, the HeLa cell line was sorted. The P3 gate identified the SP cells that excreted Hoechst 33342 and the P4 gate identified the NSP cells that were Hoechst 33342-positive (Fig. 1A). The SP cells accounted for ~1.2% of the total cell number. Following pre-incubation with verapamil for 30 min, the percentage of SP cells dropped to 0% of the total cells (Fig. 1B), which is consistent with studies that report Hoechst 33342 exclusion to be verapamil-sensitive. The SP (P3) and NSP (P4) cells were collected for subsequent experiments.

### Cell proliferation

**Figure 2:**
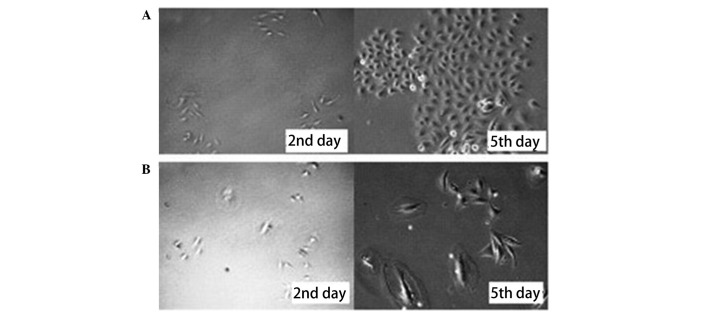
The status of the sorted (A) SP and (B) NSP cells from the HeLa cell line on the 2nd and 5th day of incubation. SP, side population; NSP, non-side population.

**Figure 3:**
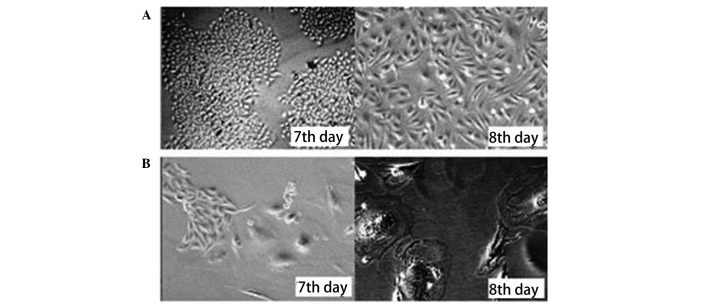
The status of the sorted (A) SP and (B) NSP cells from the HeLa cell line on the 7th and 8th day of incubation. SP, side population; NSP, non-side population.

Freshly sorted SP and NSP cells were cultured in DMEM at 37ºC in a 5% CO_2_ incubator. The growth of the cells was observed and images of the cells were captured at two, five, seven and eight days. Microscopic observation showed that the SP cells proliferated, formed colonies and spread rapidly across the culture plate. In contrast, the NSP cells grew slowly and only a fraction formed colonies. The majority of these colonies appeared swollen and disintegrated or divided several times and then disintegrated (Figs. 2 and 3).

**Figure 4:**
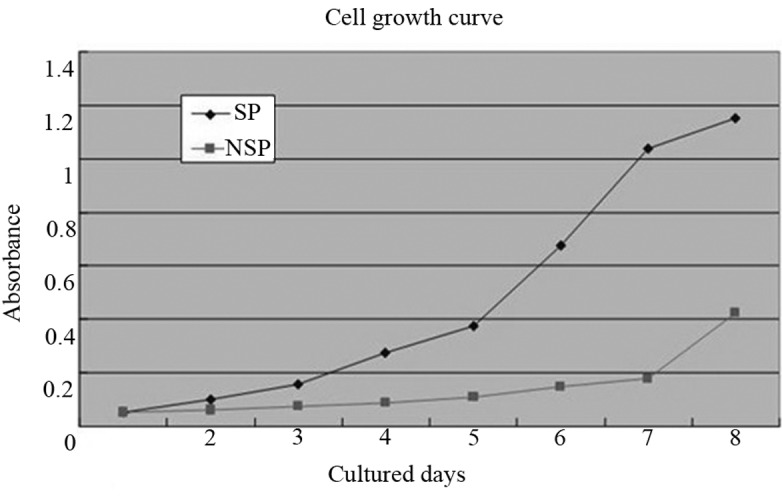
MTT cell growth curve. A significant difference was observed (P<0.05) between the SP and NSP cells. MTT, 3-(4,5)-dimethylthiahiazo (-z-y1)-3,5-di-phenytetrazoliumromide; SP, side population; NSP, non-side population.

The MTT cell growth curves showed that the SP cells entered a logarithmic growth phase within four days and a reached plateau phase within eight days. However, the NSP cells grew extremely slowly for the first seven days, following which, the growth rate accelerated. The cellular growth rates were significantly different (P<0.05) between the SP and NSP cells (Fig. 4).

### Colony-forming cell assays

**Figure 5:**
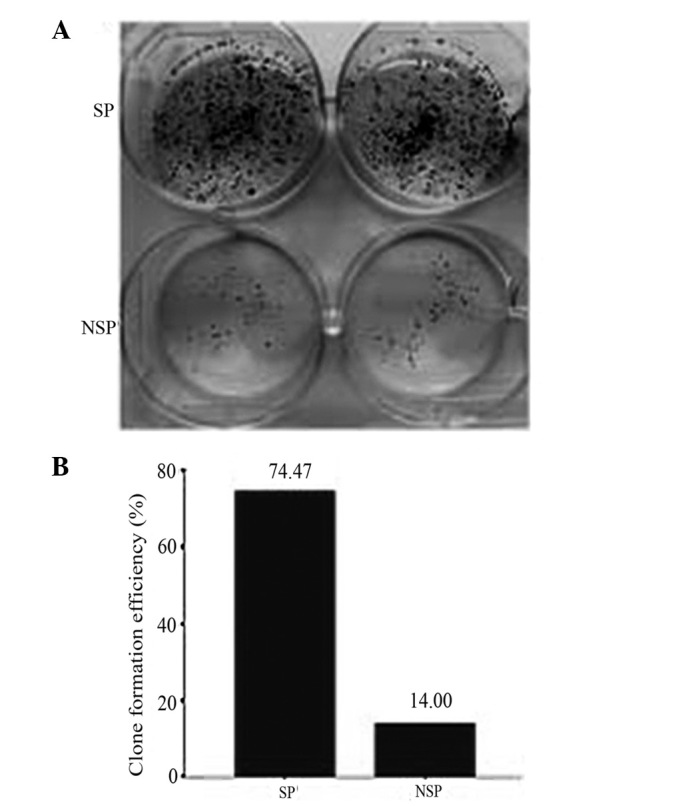
(A) SP and NSP cellular clone formation. (B) CFE for the SP and NSP cells. A significant difference (P<0.05) was observed between the SP and NSP cells. SP, side population; NSP, non-side population; CFE, clone formation efficiency.

The sorted SP and NSP cell clones expanded to >50 cells subsequent to being cultured in 6-well plates for ~10 days. The CFE of the SP and NSP cells was 74.47±4.12 and 14.00±1.19%, respectively, which represented a significant difference (P<0.05; Fig. 5). Thus, the ability of the SP cells to form clones was greater than that of the NSP cells.

### Long-term differentiation of SP and NSP cells

**Figure 6:**
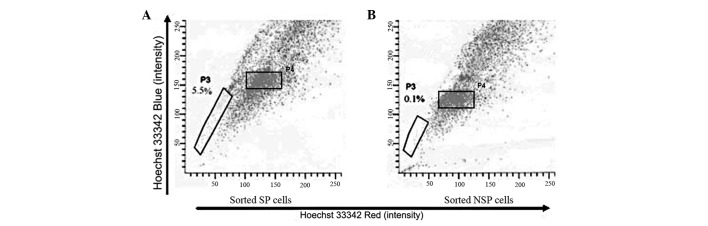
Hoechst 33342 staining of sorted SP and NSP cells analyzed using flow cytometry. (A) The percentage of SP dots that grew from the sorted SP cells was 5.5%. (B) The percentage of SP dots that grew from the sorted NSP cells was only 0.1%. SP, side population; NSP, non-side population.

Freshly sorted SP and NSP cells were cultured for three weeks, stained with Hoechst 33342 and analyzed by FACS. The percentage of SP cells that grew from the sorted SP cells was *~*5.5%, whereas the percentage of SP cells that grew from the sorted NSP cells was 0.1% (Fig. 6).

### Tumor formation assay

**Figure 7:**
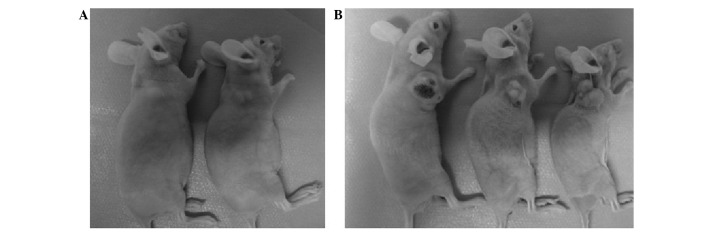
Subcutaneous tumorigenicity of the SP and NSP cells in Balb/c mice. (A) Two of the mice injected with 1×10^3^ SP cells formed tumors. (B) All three mice injected with 1×10^4^ SP cells formed tumors. SP, side population; NSP, non-side population.

Sorted SP and NSP cells were injected into the Balb/c mice and allowed to grow for eight weeks. As few as 10^3^ SP cells were sufficient for tumor formation in these mice (2/3), whereas the mice that were injected with an equal number of NSP cells produced no detectable tumors (0/3). Tumors appeared following the injection of 10^4^ SP cells (3/3). In contrast, the injection of NSP cells failed to form tumors in the Balb/c mice (0/3), with the exception of those in the group that received the highest number (10^6^) of NSP cells (Table I; Fig. 7).

### Chemotherapeutic drug sensitivity assay

**Figure 8:**
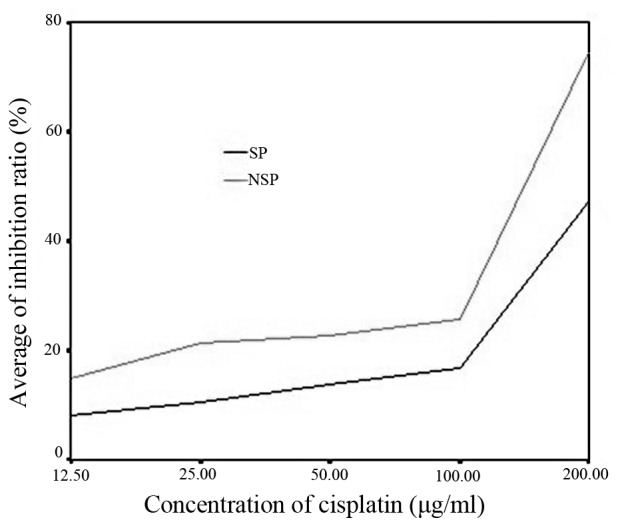
The cisplatin inhibition ratio curve for SP and NSP cells. SP, side population; NSP, non-side population.

The cell inhibition ratio was calculated for the SP and NSP cells following treatment with the various concentrations of cisplatin (Table II). The inhibition ratio curve (Fig. 8) demonstrated that cisplatin suppressed the growth of the NSP cells more than it suppressed the growth of the SP cells. Therefore, the SP cells were more tolerant to cisplatin compared with the NSP cells.

### Radiotherapy sensitivity assay

**Figure 9:**
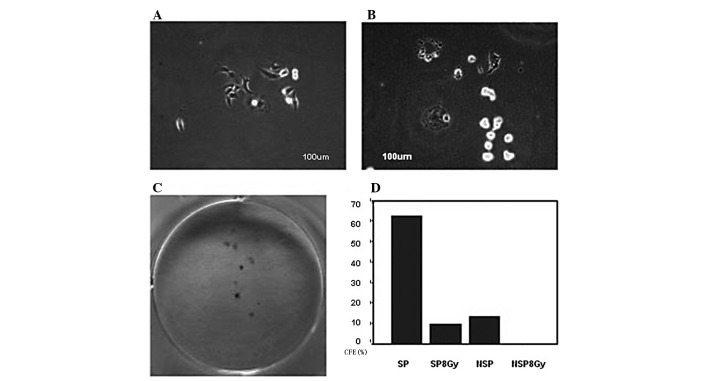
X-ray sensitivity assay for SP and NSP cells. (A) SP cells exposed to 8 Gy X-rays were cultured at one week post-exposure. The surviving cells are shown forming clones. (B) NSP cells exposed to 8 Gy X-rays were cultured, and a week later, the majority of cells had died and those that survived became swollen. (C) Clones formed from SP 8 Gy cells cultured for three weeks. (D) CFE of SP, NSP, SP 8 Gy and NSP 8 Gy cells cultured for three weeks. A significant difference (P<0.05) was observed between the SP and NSP cells. SP, side population; NSP, non-side population; CFE, clone formation efficiency.

The NSP cells were exposed to X-rays and cultured for two days. Only a small number of the exposed NSP cells survived, which then gradually became swollen and ultimately underwent apoptosis (Fig. 9B), rather than continuing to divide. A fraction of the SP cells that were exposed to X-rays died. However, the living SP cells grew slowly and formed clones within three weeks (Fig. 9A and C). The CFE of the SP cells exposed to X-rays was 9.75±2.21%, which was significantly less than that of the unexposed SP cells (63.44±2.36%; P<0.05), but significantly greater than the CFE of the NSP cells that were exposed to 8 Gy X-rays (0%; P<0.05). The CFE of the NSP cells that were exposed to 8 Gy X-rays was significantly lower than that of the NSP cells that were not exposed to X-rays (12.28±1.60%; P<0.05; Fig. 9D).

### CD133 expression in SP and NSP cells

**Figure 10:**
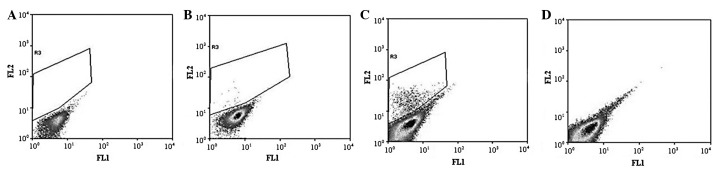
CD133 expression in common HeLa cells, SP cells and NSP cells. (A) No CD133 antibody, as control. (B) Common HeLa cells. (C) SP cells. (D) NSP cells. The drawn area represents the percentage of CD133 expression in HeLa cells. SP, side population; NSP, non-side population.

A small number of the HeLa cells expressed CD133 (~0.16% of the total cell number). No CD133 expression was detected in the NSP cells. By contrast, the fraction of CD133-positive SP cells was elevated to 3.64% (Fig. 10).

### CD44 expression in SP and NSP cells

**Figure 11:**
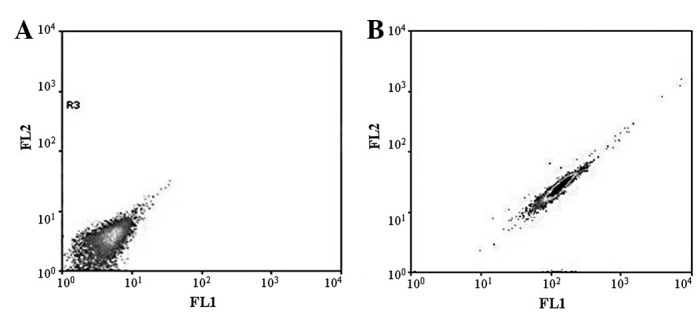
CD44 expression in HeLa cells. (A) No CD44 antibody as control. (B) Common HeLa cells reacted with the CD44 antibody. All cells were FITC-positive.

CD44 expression was positive in all the HeLa cells. There was no significant difference in CD44 expression between the SP and NSP cells (Fig. 11).

## Discussion

SP cells were first isolated from murine bone marrow by Goodell *et al* in 1996 (8). This small subset of cells were identified to express a surface marker of hematopoietic stem cells (HSCs) and were also able to rebuild the hematopoietic system of the bone marrow. Since the initial application of the SP technique in bone marrow HSCs, the method has been adapted to identify putative stem cells and progenitor cells in multiple tissues and organs, including umbilical cord blood (13), skeletal muscle (14) and mammary glands (15). SP cells possess certain intrinsic stem cell properties, as they have a long life cycle, are mostly in a relative resting state during telophase, have a high ability for self-renewal and are tolerant to chemotherapeutic drugs. In 2004, Kondo *et al*(12) first isolated SP cells from the C6 glioma cell line and suggested that the cells displayed stem cell-like characteristics. SP cells have been identified in a large variety of neoplasms. The small and rare subset of SP cells have an increased capacity to propagate tumor growth, self-renew, initiate tumor formation when xenografted into NOD/SCID mice and are particularly resistant to chemotherapeutic agents. These characteristics are shared with tumor stem cells (12,16,17).

In the present study, the SP cells were successfully sorted from the HeLa cervical adenocarcinoma cell line using FACS. The HeLa cell line contained ~1.2% SP cells, the characteristics of which were investigated. An MTT assay revealed that the rate of propagation and the colony formation capacity of the SP cells were significantly higher than that of the NSP cells in the HeLa cell line. These findings indicate that the SP cells may play a significant role in the proliferation and renewal of HeLa cells. Whereas NSP cells are mature cells that eventually undergo apoptosis, the SP cells contribute to maintaining the number of HeLa cells. This finding is consistent with other studies (12,16,17) and suggests that the SP cells from the HeLa cell line have a strong capacity for propagation and colony formation. In the tumor formation assay of the present study, as few as 10^3^ SP cells were sufficient for tumor formation in the Balb/c mice. The tumorigenic ability of the SP cells was 1,000 times higher than that of the NSP cells. Therefore, the SP cells were significantly more tumorigenic than the NSP cells. One of the most crucial functions of stem cells is self-renewal. In the present study, the SP cells showed a higher survival ability compared with the NSP cells. Stem cells grow through two mechanisms, symmetrical division and an asymmetrical second division (18–21). The present data revealed that the percentage of the SP cells within the sorted HeLa cells was ~5.5% and that the remaining cells were NSP cells, indicating that SP cells in culture are able to self-renew and generate SP and NSP progeny. However, the NSP cells that are produced are mature cells, which are terminally differentiated. Under the same culture conditions, the cells produce NSP cells only. In contrast to the SP cells, NSP cells undergo apoptosis following several generations of division.

According to the CSC theory, a small subpopulation of cancer cells may have the capacity to tolerate the aggressive insults of radiotherapy and chemotherapy. These cells may be responsible for cancer reoccurrence. CSCs may be able to re-establish cancer even if the majority of the cancer cells are killed (22). In the present study, the examination of the resistance to radiotherapy and chemotherapy demonstrated that none of the NSP cells were able to survive for more than a few days following exposure to 8 Gy X-rays. Although the CFE of the SP cells that were exposed to X-rays was lower than that of the unexposed SP cells, the exposed SP cells demonstrated a significantly higher CFE than the exposed NSP cells. Thus, SP cells are more resistant to radiotherapy than NSP cells and have the characteristics of CSCs. An accepted hypothesis of chemoresistance proposes that standard therapies fail to target tumor progenitors that express normal stem cell phenotypes (23). In the present study, SP cells were identified to be more resistant to cisplatin, indicating a role for these cells in chemoresistance in cervical cancer. It has been reported that high levels of the ABC transporter protein, ABCG2, enhance the efflux capacity of SP cells for Hoechst 33342 dye and for lipophilic anti-cancer drugs, including those used for cervical cancer. If the ABC transporter is blocked, cells become sensitive to drugs (24). In the present study, western blot analyses showed that although ABCG2 was overexpressed in the SP cells, the NSP cells hardly expressed the protein (data not shown). This finding demonstrates that the mechanism underlying chemoresistance is likely to involve the overexpression of ABCG2. Evidence obtained in the present study has demonstrated that the SP cells that were sorted from the HeLa cell line present a strong capacity for cell proliferation, tumor formation, self-renewal, differentiation and resistance to radiotherapy and chemotherapy, which are typical CSC characteristics.

CD133 is a membrane glycoprotein that has generated much interest in the stem cell field. The glycoprotein was originally discovered in hematopoietic stem or progenitor cells and CD133 expression has been confirmed in CSCs from the colon (25), pancreas (26), liver (27) and larynx (28). CD133 is therefore considered to be a marker for CSCs. In the present study, CD133 was expressed by ~3.64% of the SP cells, but none of the NSP cells. Although the ratio of CD133^+^ SP cells is low, CD133 may be considered as a surface marker for cervical adenocarcinoma stem cells. Thus, SP cells are enriched with tumorigenic stem-like cancer cells, but are not equal to CSCs. Furthermore, SP cells differentiate into NSP progeny during long-term culture. CSCs expressing CD133 that have been isolated from gliomas are more resistant to radiotherapy than CD133^−^ tumor cells (29). The role of CD133 expression in SP cells from the HeLa cell line, particularly as a biomarker for cervical cancer, requires further investigation.

CD44 is considered to be a biomarker for breast (3) and ovarian (30) CSCs. In the present study, all HeLa cells were observed to express CD44, as determined by flow cytometry. CD44 is therefore not considered to be a biomarker for cervical adenocarcinoma stem cells.

In summary, the SP cells that were isolated from the HeLa cell line demonstrated enhanced self-renewal, a high proliferation potential *in vitro*, a strong ability to form tumors *in vivo* and a high resistance to radiotherapy and chemotherapy, which are all properties of CSCs. In cervical cancer, as in other cancers, the characterization of CSCs may allow the development of new treatments that are specifically targeted against this critical population of SP cells, particularly against their ability to self-renew, which may result in more effective therapies.

## Figures and Tables

**Table I:** *In vivo* tumor formation ability of the SP and NSP cells in Balb/c mice.

	Quantity of transplanted cells			
	
Cell type, n/total	1×10^3^	1×10^4^	1×10^5^	1×10^6^
SP cells	2/3	3/3	3/3	3/3
NSP cells	0/3	0/3	0/3	2/3

SP, side population; NSP, non-side population.

**Table II:** Cisplatin sensitivity of SP and NSP cells.

Cisplatin concentration (μg/ml)	Inhibition ratio		P-value

	SP cells	NSP cells	
12.5	8.22±2.54	14.86±0.65	<0.05
25.0	10.58±3.84	21.28±4.52	<0.05
50.0	13.74±3.19	22.61±2.59	<0.05
100.0	16.67±2.17	25.76±4.75	<0.05
200.0	47.30±3.66	74.51±1.88	<0.05

SP, side population; NSP, non-side population. Data presented as mean ±SD.
